# Local Research Catalyzes National Surgical Planning: Comment on "Global Surgery – Informing National Strategies for Scaling Up Surgery in Sub-Saharan Africa"

**DOI:** 10.15171/ijhpm.2018.78

**Published:** 2018-08-08

**Authors:** Micah G. Katz, Raymond R. Price, Jade M. Nunez

**Affiliations:** Center for Global Surgery, University of Utah School of Medicine, Salt Lake City, UT, USA.

**Keywords:** Global Surgery, Africa, Systems Approach, National Surgical Plans

## Abstract

In 2015 the Lancet Commission on Global Surgery (LCoGS) argued that surgical care is important to national health systems along with the economic viability of countries. Gajewski and colleagues outlined how the Commission’s blueprint has been implemented in sub-Saharan Africa, including two funded research projects that were integrated into national surgical plans. Here, we outline how the five processes proposed by Gajewski and colleagues are critical to integrate research, policy, and on-the-ground implementation. We also propose that, moving forward, the most pressing adjunct in many low- and middle-income countries (LMICs) may be a better characterization of rural surgical practices through rigorous research along with models that enable lessons to inform national policy.

The accompanying article by Gajewski and colleagues^1^ is a thoughtful exploration of how *Global Surgery 2030: evidence and solutions for achieving health, welfare and economic development*^2^ published by the Lancet Commission on Global Surgery (LCoGS) has influenced national surgical and anesthesia plans in sub-Saharan Africa. It focuses specifically on the need for collaboration between ministries of health and systems research stakeholders in the development of national surgical plans.

The ultimate goal in the building of surgical and anesthesia capacity is to improve the accessibility and quality of services worldwide. This requires a nuanced understanding of the contexts in which care is currently delivered. Healthcare transformation can generally be achieved by a “top-down” strategy – often in the form of national surgical plans – or “bottom-up” – grassroots efforts that often uses self-organization to bring change. Using either approach, the provision of medical care without the input of research or evidence-based guidelines runs the risk of being inefficient or ineffective. Conversely, research and policies that are not grounded in implementation science may be limited to academic exercises. The coordination of “top-down” or “bottom-up” strategies and the interaction of research, policy, and surgical and anesthesia delivery is paramount, but lacking in many low-resourced areas. The LCoGS offered a blueprint for coordinating the efforts of these groups.

*Global Surgery 2030*, however, is a product of the same problem it addresses: more research is needed to inform national programs. Only 4.1% of global health research is surgical, of which only 4.3% is relevant to underserved populations.^3^ Reflecting the dearth of surgical research, few of the world’s researchers are based in low- and middle-income countries (LMICs) and even fewer focus on health services and systems research.^4,5^ Of particular concern, there is little literature that addresses peri-operative systems including operation room management, nursing and intensive care. While overall research in LMICs is limited, our understanding of surgical systems in rural areas and district hospitals is especially lacking.

A recent study by members of the Ghana Hernia Society demonstrates the importance of research that includes district-level hospitals.^6^ In a retrospective review of over 8000 inguinal hernia repairs in northern Ghana, the majority (84%) are repaired in district hospitals by non-surgeon physicians (66%). In contrast, the majority of research on hernia repair, such as the cost-effectiveness^7^ and the safety of low-cost or sterilized mosquito net mesh,^8,9^ is based in large, regional or teaching hospitals and performed by surgeons. They also found that only 37% of hernia repairs were performed under local anesthesia and that majority of physicians and anesthesia providers were unfamiliar with evidence and guidelines that favor this form of anesthesia. To close the gap between research and clinical practice, the Ghana Hernia Society has already begun a project to provide hernia-specific training to non-surgeon physicians^10^ and anesthesia providers and is involved with ongoing research on the effectiveness of that training and subsequent outcomes in district hospitals.

For the Ghana Hernia Society to affect national policy, they must garner support from key stakeholders including the Ministry of Health. In this way, they may complement their efforts through integration with “top-down” national surgical plans. This lesson is highlighted by the country’s past efforts to improve trauma care.^11^ In 1997, a group of surgeons created a 20-page proposal to strengthen the trauma system in Ghana. Without buy-in from the Ministry of Health, the proposal “gathered dust.” Meanwhile, research and the media documented the ongoing tragedy of trauma deaths in the country. Growing public outcry along with international guidelines fostered a workshop that brought together researchers, clinicians, the Ministry of Health, and other stakeholders. The resulting plan and corresponding document, *Strengthening Care for Injury Victims: Recommendations for a National Policy,* catalyzed drastic improvements in trauma care in Ghana including: a national ambulance service,^12^ trauma education courses,^13^ an emergency medicine training program, and generally improved trauma care capacity.^14^ The Ghanaian trauma system, guided by research, shaped by policy, and implemented by clinicians demonstrates how a coordinated surgical and anesthesia system can effect change.

The LCoGS offered a compelling argument for the importance of expanding access to safe and affordable surgical and anesthesia care along with a blueprint for moving toward this target. Now three years following the publication of *Global Surgery 2030*, its recommendations remain critically relevant and have shaped much global surgery research since publication. How has research guided by the recommendations of the LCoGS affected patient care? And how has such research shifted the conversation and added nuance? For example, how should a country reconcile high rates of timely access to Bellwether procedures faced with the reality of hospitals lacking basic infrastructure necessary to provide safe surgical and anesthesia care?^15^ How should non-physician clinicians be factored into the surgical and anesthesia workforce density? Are perioperative mortality rates sufficient without basic risk adjustment?^16^

Gajewski and colleagues build on this conversation, and they frame their argument through the LCoGS’s recommendations. To integrate research, policy, and on-the-ground implementation, the authors recommend five elements in the building of surgical systems that involve national leaders, researchers, approved surgical clinicians, and feedback loops to allow continuous adaptation. Vital to this group, they include clinicians at the level of district hospitals. This kind of integrated surgical ecosystem is exactly what is needed to bridge the gap between the boots on the ground and policy-makers.

The authors end by citing their own experience in scaling up national surgical services in Zambia, an exceptional example of how systems research can help shape national policy. In their case, a ‘serendipitous’ confluence of funding and events make for a perfect case study and an important step toward creating a model that is low-cost, scalable, and adaptable to multiple settings. Gajewski and colleagues highlight the experience of non-physician clinicians in Zambia, who provide care for those who would otherwise not have access in rural settings.^17^ In a qualitative study of 43 interviewees, their group explored the benefits of surgical task shifting, but they also described the limitations of and difficulties faced by non-physician clinicians. Gajewski and colleagues then bridge these lessons into recommendations for stakeholders and policy-makers. This research, along with related models for supervision, have subsequently been expanded and incorporated into the national surgical plan.

Not all countries in sub-Saharan Africa, however, will have access to international support and funding. Further, each country faces a unique set of ethnic, geographic, security, political, religious, and social problems, the influence of foreign academicians may not always be needed or welcomed. Indeed, an effective national surgical plan is sometimes not feasible. The article by Gajewski and colleagues^1^ highlights the topic of research informing national surgical plans, however, other models for healthcare transformation should also be pursued. Private-public partnerships, non-governmental organizations, educational paradigms, among others are all part of complementary solutions. We must reflect the sentiment that no ‘one size fits all,’ and that learning by doing and embracing error is important. We are in uncharted territory and even the robust recommendations set forth by the LCoGS are bound to evolve. However, well-designed research and evidence-based interventions are an essential element to improve healthcare systems.

The global health community is turning its focus away from narrow, disease-specific objectives and toward strengthening entire health systems. Accordingly, the LCoGS has effectively argued for the importance to a health system of essential surgical care as well as its economic viability. Gajewski and colleagues have done well to detail an inclusive model that includes five critical processes for how stakeholders can work together to realize the LCoGS recommendations. Moving forward, the most pressing adjunct in many LMICs may be a better characterization of rural surgical practices through rigorous research along with models that enable lessons to inform national policy. “One size does not fit all,” but we all benefit from real world lessons based on experiences in Zambia. National surgical plans need to be informed by research that characterizes the needs of rural and the most underserved populations.

## Ethical issues

Not applicable.

## Competing interests

Authors declare that they have no competing interests.

## Authors’ contributions

All authors co-wrote and edited the commentary
